# The Role of Neutrophil Extracellular Traps in Periodontitis

**DOI:** 10.3389/fcimb.2021.639144

**Published:** 2021-03-18

**Authors:** Jingyi Wang, Yucong Zhou, Biao Ren, Ling Zou, Bing He, Mingyun Li

**Affiliations:** State Key Laboratory of Oral Diseases, West China Hospital of Stomatology, National Clinical Research Center for Oral Diseases, Sichuan University, Chengdu, China

**Keywords:** periodontitis, neutrophil extracellular traps, innate immunity, NETosis, inflammation

## Abstract

Periodontitis is a chronic, destructive disease of periodontal tissues caused by multifaceted, dynamic interactions. Periodontal bacteria and host immunity jointly contribute to the pathological processes of the disease. The dysbiotic microbial communities elicit an excessive immune response, mainly by polymorphonuclear neutrophils (PMNs). As one of the main mechanisms of PMN immune response in the oral cavity, neutrophil extracellular traps (NETs) play a crucial role in the initiation and progression of late-onset periodontitis. NETs are generated and released by neutrophils stimulated by various irritants, such as pathogens, host-derived mediators, and drugs. Chromatin and proteins are the main components of NETs. Depending on the characteristics of the processes, three main pathways of NET formation have been described. NETs can trap and kill pathogens by increased expression of antibacterial components and identifying and trapping bacteria to restrict their spread. Moreover, NETs can promote and reduce inflammation, inflicting injuries on the tissues during the pro-inflammation process. During their long-term encounter with NETs, periodontal bacteria have developed various mechanisms, including breaking down DNA of NETs, degrading antibacterial proteins, and impacting NET levels in the pocket environment to resist the antibacterial function of NETs. In addition, periodontal pathogens can secrete pro-inflammatory factors to perpetuate the inflammatory environment and a friendly growth environment, which are responsible for the progressive tissue damage. By learning the strategies of pathogens, regulating the periodontal concentration of NETs becomes possible. Some practical ways to treat late-onset periodontitis are reducing the concentration of NETs, administering anti-inflammatory therapy, and prescribing broad-spectrum and specific antibacterial agents. This review mainly focuses on the mechanism of NETs, pathogenesis of periodontitis, and potential therapeutic approaches based on interactions between NETs and periodontal pathogens.

## Introduction

Polymorphonuclear neutrophils (PMNs) have a pivotal role in infection, inflammation, and innate immunity. The outcome of interactions between neutrophils and oral bacteria is an important determinant of oral health. PMNs from the peripheral blood migrate through oral mucosal tissues to tissue damage, inflammation, and infection sites in the oral cavity and become oral polymorphonuclear neutrophils (oPMNs). The number of oPMNs is positively correlated with the severity of oral inflammation (Moonen et al., 2019). oPMNs play their roles by several mechanisms, such as phagocytosis, degranulation, and production of reactive oxygen species (ROS) and neutrophil extracellular traps (NETs). Phagocytosis is a mechanism to eliminate foreign pathogens and cell debris. Degranulation helps to create an antibacterial environment. The process by which activated neutrophils produce reactive oxygen species is known as a respiratory burst. The body uses reactive oxygen species to modify and inhibit the function of other molecules for signal transmission and antimicrobial activity (Amulic et al., 2012).

NETs are reticular structures secreted by activated neutrophils. Different stimuli can lead to NETs with specific compositions. NETs are composed of decondensed DNA and antimicrobial proteins, and both nuclear and mitochondrial DNA can be found in NETs (Li et al., 2020). NETs proteins include histones, human neutrophil elastase (NE), myeloperoxidase (MPO), cathelicidins, defensins, actin lysozyme, bactericidal permeability-increasing protein, and human peptidoglycan-recognition protein S (Brinkmann et al., 2004; Pires et al., 2016). NETs have both pro-inflammatory and anti-inflammatory effects and are released outside neutrophils to play a pro-inflammatory role in immobilizing and destroying extracellular pathogens. Protein-arginine deiminase 4 (PAD4) is an important target of neutrophils in the formation of NETs. A study using PAD4 inhibitors to modulate phenotypes crucial for lupus pathogenesis and disease activity suggested that NETs have pro-inflammatory functions (Knight et al., 2013). However, although NETs are involved in Papillon-Lefevre syndrome (PLS), patients with sufficient NETs formation have been reported to be more seriously ill (Schauer et al., 2014). The mechanisms of the anti-inflammatory process have not been explained yet. However, a study showed that aggregated NETs could resolve inflammation by the proteolysis of cytokines and chemokines (Hahn et al., 2019). Current evidence also suggests that NETs contribute to many diseases like thrombosis (Kimball et al., 2016), atherosclerosis (Franck et al., 2018), autoimmune diseases (Frangou et al., 2019a; Frangou et al., 2019b), sepsis (Denning et al., 2019), and periodontitis (Magan-Fernandez et al., 2020).

Severe periodontitis affects approximately 10% of the world population (Frencken et al., 2017), indicating its importance, which provides continuous motivation for correlational studies. Periodontitis is a multifactorial inflammatory disease that affects periodontal supporting tissues, including the gingiva, periodontal ligament, and alveolar bone, collectively known as the periodontium (Pihlstrom et al., 2005). The initiating factor for periodontitis is generally considered to be the accumulation of dental microbial biofilms at and below the gingival margin (Loe et al., 1965). Periodontal dysbiosis is a pivotal factor in the development of periodontitis (Rosier et al., 2014; Lamont and Hajishengallis, 2015). Dysbiotic microbial communities can elicit persistent immune responses. Currently, research mainly focuses on the dysbiosis of periodontal microbiota and host factors to prevent and treat periodontitis (Kilian et al., 2016; Rosier et al., 2018). PMNs are deeply involved in these mechanisms. The homeostasis of oPMNs is significant. Both excess and deficiency of oPMNs can lead to periodontitis (Ye et al., 2011; Moutsopoulos et al., 2014; Sorensen et al., 2014; Cortes-Vieyra et al., 2016; Makkawi et al., 2017; Silva et al., 2019).

Similarly, it is evident that responses to biofilms are species-specific and might support either the maintenance of oral health or pathogenesis of periodontitis, depending on the species (Hirschfeld et al., 2017; Mikolai et al., 2021). Besides, in periodontitis cases, oPMNs are highly activated, live longer, and are recruited more than usual (da Silva et al., 2017; Tonetti et al., 2017; Ebersole et al., 2018). The quality and quantity of the host’s inflammatory and immune responses are also significant in periodontitis (Hajishengallis, 2014a; Lamont et al., 2018). Over time, many microbes have evolved mechanisms to evade NETs. Some bacteria can express nucleases to degrade DNA and protein of NETs (de Buhr et al., 2014; Juneau et al., 2015; Doke et al., 2017; Stobernack et al., 2018; Bryzek et al., 2019). Moreover, some pathogens can regulate the NET formation and even exploit inflammation to thrive under dysbiotic conditions.

This review aims to describe the formation and removal of NETs and how they work in periodontitis. Additionally, the review discusses the homeostasis of periodontal pathogens and neutrophils and the relationship between them in the pathological process of periodontitis in detail. Finally, the review explains how bacteria escape NETs and which potential therapeutic approaches are used to treat periodontitis.

## NETs

### Induction

Many stimuli can induce PMNs to produce NETs, such as microorganisms, inflammatory cytokines, and other agents like calcium ionophores and HOCl (Brinkmann, 2018). As a common component in the cell wall of Gram-negative bacteria, lipopolysaccharides (LPS) can induce NETs as well (Aquino-Martinez et al., 2020). Different activators mediate different NETs, forming signaling pathways. Kenny et al. (2017) found that among five inducers, phorbol 12-myristate 13-acetate (PMA), *Candida albicans*, and group B *Streptococcus* (GBS) shared a common pathway for NET formation. The calcium ionophore A23187 elicits NET release using an alternative pathway. Similarly, different signaling pathways are affected by different interferences. Cigarette smoke extract (CSE) disturbs the NET formation process by interfering with neutrophil degranulation and ROS generation (Hosseinzadeh et al., 2016). ROS contribute to the NET formation by helping nuclear membrane breakdown. PMA is a protein kinase C (PKC) agonist, while HOCl activates NET formation downstream of PKC. An *in vitro* study demonstrated that after pre-treatment of CSE, PMA-induced NET formation, but not HOCl-induced NET formation, was affected (White et al., 2018).

Whether or not PMNs choose NETs to kill invading organisms depends on the size of stimuli and the virulence factors of pathogens. As for the size, NET formation specializes in eliminating larger bacteria, while phagosomes take up the smaller ones (Papayannopoulos, 2018). When PMNs are stimulated by bigger microorganisms, NE will be transferred to the nucleus to start the NETotic cell death (Metzler et al., 2014). On the contrary, once phagocytosis begins, NE is removed from the nucleus, and chromatin decondensation is inhibited, meaning that NETs are not formed. It is the same with sterile stimulus. Compared with urate microaggregates, larger and needle-shaped urate crystals can more easily induce NETotic cell death (Pieterse et al., 2016). Small bacteria, too, can induce NET formation under some conditions. Certain small bacteria induce NET formation by forming large aggregates, e.g., *Mycobacterium bovis* aggregates (Branzk et al., 2014). In the presence of IgA, both increased phagocytosis and NET release could be observed (Aleyd et al., 2014). Microbial virulence factors could affect NET formation by interfering with the maturation of phagocytes (Johnson and Criss, 2013), altering neutrophil cell biology (Knodler et al., 2010) and promoting the association of NET components (Spaan et al., 2013). Moreover, by expressing invasion, which enhances the ROS burst, *Yersinia pseudotuberculosis* induce NET formation successfully (Gillenius and Urban, 2015).

In addition to the size and virulence factors of inducers mentioned above, NET formation is also impacted by the oxygen content, pH, and bicarbonate of the internal environment. Changes in extracellular pH alter intracellular pH rapidly. Higher pH of neutrophils increases NADPH oxidase 2 (NOX)-dependent NET formation by stimulating NOX-mediated ROS production and histone H4 cleavage in NET formation (Khan et al., 2018). In contrast, low pH suppresses NET formation. There is a triangular relationship between CO_2_, bicarbonate, and pH. Bicarbonate dose-dependently induces intracellular alkalinization and intracellular increase in calcium content. High calcium content makes it easier for neutrophils to release NETs. The average pH of the periodontal pocket was near neutral (Eggert et al., 1991). Glucose and glycolysis are inevitable during NET formation (Rodriguez-Espinosa et al., 2015). The NET formation was observed to be induced by acute glucose fluctuations (Miyoshi et al., 2016).

Although respiratory burst occurs in all PMNs after being stimulated, which means they can produce NETs, not all activated PMNs end up producing NETs (Sorensen and Borregaard, 2016; Deniset and Kubes, 2018). The exact mechanism of this phenomenon is not very clear. However, studies have shown that PMNs fall into different subtypes (Deniset and Kubes, 2018), with different biochemical reactions. For instance, PMNs are thought to consist of two types depending on the expression of granule protein olfactomedin 4 (OLFM4). OLFM4^+^ and OLFM4^–^ PMNs can produce different qualities of NETs (Welin et al., 2013). OLFM4 was found only in NETs formed by OLFM4^+^ PMNs. Only mature PMNs can release NETs upon IFN-α/γ priming and following stimulation with the complement factor C5 (Martinelli et al., 2004).

### Different Types of NET Formation

**Figure 1:**
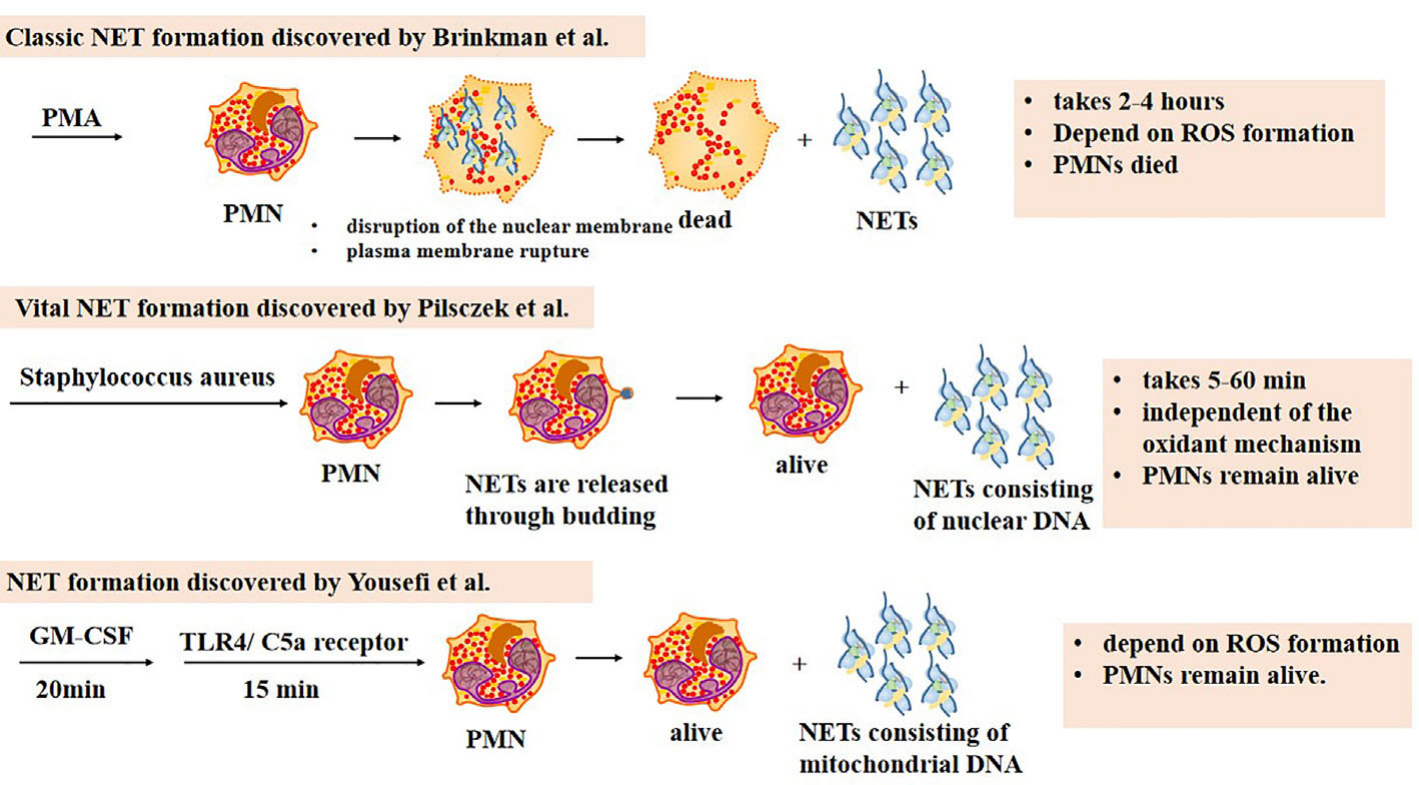
Three types of NET formation. The classic NET formation pathway starts with chromatin decondensation, after which the nuclear membrane disrupts and the decondensed chromatin mixes with neutrophil peptides. NETs are released outside neutrophils once the plasma membrane ruptures, which means neutrophils are dead. The whole process lasts 2–4 h, dependent on ROS formation. The second one is named vital NET formation, which indicates that PMNs are alive after the formation. In this pathway, NETs are released to the extracellular space by budding. It takes 5–60 min to complete the process, independent on ROS formation. Only NETs consisting of nuclear DNA can be harvested during the process. The third NET formation pathway needs 20 min stimulation of granulocyte/macrophage colony-stimulating factor (GM-CSF) and subsequently 15 min stimulation of short-term toll-like receptor 4 (TLR4) or complement factor 5a (C5a) receptor stimulation. NETs consisting of mitochondrial DNA are released. PMNs remain alive after the whole process which depends on ROS formation.

To date, three types of NET formation processes have been reported (**Figure 1**). The most classic and best-described approach was proposed by Brinkman in 2004 (Brinkmann et al., 2004), during which nuclear DNA is released along with the destruction of PMN nuclear membrane. In this process, PMNs recognize the stimulus first. Then PMNs activate PAD4, which elicit histone deamination and release ROS using calcium pools inside and outside the cells (Gupta et al., 2014). The autosomes and heterosome separate subsequently, leading to the disappearance of the typical lobulated nucleus. Cytoplasm and nucleus mix as the nuclear membrane is lysed. Finally, the cell membrane breaks down, and NETs are released to the extracellular environment. This process is promoted by ROS and lasts for 2–4 h. Unlike apoptosis and necrosis, it is a kind of procedural death (Fuchs et al., 2007).

It is worth mentioning that the term “NETosis” has previously been used to represent the formation of NETs and the death of PMNs, indicating that the death of PMNs is inevitable. However, reports reveal that not all the PMNs are doomed to die during the course. As a result, researchers suggest using the term “NET formation” instead of “NETosis” (Boeltz et al., 2019). The term “NETotic cell death” has also been used in the literature (Galluzzi et al., 2018).

In 2009, Yousefi et al. (2009) reported that PMNs could generate NETs with mitochondrial DNA. Firstly, PMNs are primed with granulocyte/macrophage colony-stimulating factor (GM-CSF) for 20 min. Then short-term toll-like receptor 4 (TLR4) or complement factor 5a (C5a) receptor stimulation is applied for 15 min. Unlike the classic formation, PMNs stay alive after NETs are released. The process takes less time and requires less stimulus. ROS formation is also required for this kind of formation.

In 2012, another special NET formation was discovered to be induced by *Staphylococcus aureus*. During this procedure, nuclear DNA is stored in vesicles and released through budding without nuclear membrane dissolution (Pilsczek et al., 2010). Mitochondrial DNA barely constitutes NETs. Finally, the chromatin is released out of the cell. The whole process takes 5–60 min, which is really fast compared with the classic one. Moreover, this type of NET formation is independent of the oxidant mechanism. Similar to the one reported by Yousefi, PMNs remain alive.

Expect from PMNs, other cells, too, can release web-like structures, such as mast cells (MCs) and eosinophils. MCs are the first to be reported to produce extracellular structures that have components similar to NETs, including DNA, histones, tryptase, and antimicrobial peptides (AMPs) (von Kockritz-Blickwede et al., 2008). Primed with IL-5 or IFN-γDNA, eosinophils release web-like structures that consist of mitochondrial DNA (Yousefi et al., 2008). This process does not involve cell death and is very rapid.

### Functions of NETs

First of all, NETs can fight against bacteria, parasites, fungi, and viruses. NETs trap pathogens and limit their spread by binding their cationic components to the bacterial anionic surface (Branzk et al., 2014). The antibacterial activity of NETs depends on the components of NETs, with intrinsic antimicrobial activity. As mentioned above, there are many kinds of AMPs among the proteins constituting NETs. AMPs can fight against not only bacteria but also fungi and parasites (Sierra et al., 2017). In addition, the DNA of NETs can play an antibacterial role. Chromatin in animals, plants, and unicellular eukaryotes are involved in the immune response (Zhang et al., 2016). The phosphodiester backbone of NETs DNA provides the ability to bind to cation, destroying membrane integrity and lysing bacterial cells (Halverson et al., 2015). Pentraxin 3 (PTX3), a circular multimeric glycoprotein, is one of the proteins in NETs. It displays elegant microbial effects by recognizing pathogens, regulating complements, and associating with other NET components. Growing evidence shows that PTX3 could regulate extracellular histones, too (Yost et al., 2016).

NETs can both promote and reduce host inflammation. Of the neutrophilic inflammation models, mice deficient in NETs developed more severe inflammation, which could be relieved by adoptive transfer of NETs (Schauer et al., 2014). Further studies show that aggregated neutrophil extracellular traps resolve inflammation by the proteolysis of cytokines and chemokines (Hahn et al., 2019). Under the high density of PMNs, serine proteases, part of NETs components, can degrade cytokines and chemokines, indicating their anti-inflammatory activity.

When NETs occur in the wrong place at the wrong time and last too long, they can lead to various diseases, such as Rheumatoid Arthritis, lupus, and vasculitis, by destroying body tissues and prolonging the inflammatory response.

In addition, NETs can participate in autoimmunity by activating the complement system *via* alternative or classic pathways (Yuen et al., 2016). At present, ever-increasing evidence proves that the complement system, agglutination, and NET formation are inextricably related. More attention should be paid to the mechanisms of NET-related diseases (de Bont et al., 2019).

### Measurement and Removal of NETs

NETs can be active for days (Brinkmann et al., 2004). The excessive NET formation will damage body tissues since NETs contribute to inflammatory and autoimmune diseases. Hosts continuously remove NETs through various mechanisms to maintain proper extracellular concentrations of NETs. A study on vascular occlusion showed that deoxyribonuclease I (DNase-1), along with DNase1-like 3 (DNase1L3), could degrade NETs in the circulation (Jiménez-Alcázar et al., 2017). Macrophages could secrete DNases to degrade the DNA component of NETs and then uptake the residual part by endocytosis. Of note, no pro-inflammatory reaction occurs during this process (Haider et al., 2020). Although DNase degrades the DNA in NETS, the peptide that forms NETS might be free in tissue fluid and cause damage to cells; therefore, it is necessary to find other ways to degrade the peptide (Kolaczkowska et al., 2015). The cytosolic exonuclease TREX1 in macrophages can degrade NETs directly, while DNase1L3 mediates degradation by digesting the nuclear chromatin of NETs (Lazzaretto and Fadeel, 2019).

Through technical devices, we can further understand the microstructure and formation mechanisms of NETs. Therefore, a positive correlation between the circulating levels of NETs and periodontitis severity has been validated (Kaneko et al., 2018). The NET levels could be influenced by periodontal treatment (White et al., 2016). These findings indicate the potential of NETs to be a biomarker, which can be used as a diagnostic index and help evaluate the therapeutic effects of periodontal treatments.

Fluorescence microscopy (Doke et al., 2017), confocal microscopy, transmission electron microscopy (TEM), and scanning electron microscopy (SEM) (Vitkov et al., 2009) are all efficient methods for validating NET formation and visualizing NET structures. However, microscopy alone cannot be used for quantification. Fluorometric detection of DNA is frequently used to quantify NET release or degradation in plasma (White et al., 2016; Doke et al., 2017; Obama et al., 2019; Moonen et al., 2020). There are other DNA releasing activities, such as necrosis, which would confound the results. Therefore, AMPs associated with the DNA backbone would be co-labeled. Citrullinated histones 3 (CitH3), MPO, NET-associated MPO-DNA complexes, histone H1, CD-177, NE, and cathepsin-G (Hirschfeld et al., 2015; Kaneko et al., 2018; Magan-Fernandez et al., 2019) are supplementary NET markers for determining NET-DNA sequence. Although fluorometric detection is commonly used for quantifying NETs, the specificity and objectivity need to be verified, since the results are obtained in photographic form. Some researchers mistakenly thought they had found a new type of NET formation, because of using incorrect detection methods. Thus, a gold standard for detecting NETs is required.

## Periodontitis

Periodontitis is a chronic multifactorial inflammatory disease that compromises the integrity of tooth-supporting periodontal tissues (Pihlstrom et al., 2005). Characterized by progressive loss of attachments and alveolar bone, which eventually leads to tooth loss, periodontitis also has adverse impacts on the appearance, mastication, and systemic health (Hajishengallis, 2015). Traditionally, the accumulation of dental microbial biofilm at and below the gingival margin has been considered the main etiologic factor for periodontitis (Loe et al., 1965). However, recent advances (Rosier et al., 2014; Lamont and Hajishengallis, 2015) suggest that the emergence and persistence of dysbiotic periodontal microbial communities are the pivotal etiologic factors of periodontitis. The term “dysbiosis” signifies an imbalance in the relative frequency rather than the appearance of new species (Abusleme et al., 2013). *Porphyromonas gingivalis* (*P. gingivalis*), *Treponema denticola*, and *Tannerella forsythia* have been considered keystone microbial species to induce periodontitis (Socransky and Haffajee, 2005). *P. gingivalis* alone cannot cause periodontitis in germ-free mice, indicating that *P. gingivalis*-induced periodontitis requires the presence of the commensal microbiota (Hajishengallis et al., 2011). The microbial dysbiosis within the subgingival plaque biofilm elicits immune responses. Then, under the interactions of dysbiotic microbial communities and host immunity, the periodontium undergoes a continuous inflammatory state. Furthermore, inappropriate host immune responses result in tissue damage. The quality and quantity of hosts’ inflammatory and immune responses are significant to periodontitis (Hajishengallis, 2014a; Lamont et al., 2018), modified by environmental factors, systemic health status, and genetic characteristics (Nociti et al., 2015; da Silva et al., 2017; Tonetti et al., 2017; Ebersole et al., 2018; White et al., 2018). For instance, environmental factors, like cigarette smoke (Nociti et al., 2015; White et al., 2018), can lead to increased susceptibility, greater severity, and faster progression of periodontal disease. Further, a variety of systemic inflammatory diseases have been suggested to have epidemiological, clinical, pathological, and bi-directional associations with periodontitis, including diabetes (Ussar et al., 2016), atherosclerotic cardiovascular diseases (Beukers et al., 2017; Carrizales-Sepulveda et al., 2018; Sanz et al., 2020), rheumatoid arthritis (RA) (Leech and Bartold, 2015; Konig et al., 2016), and dementia (Daly et al., 2018). Besides, older people are more vulnerable to periodontitis, which might be attributed to immune senescence and deteriorating health due to gradual aging (Tonetti et al., 2017; Ebersole et al., 2018).

## Oral PMNs

Circulatory PMNs (cPMNs) can be recruited to every site needed (Deniset and Kubes, 2016; Kubes, 2018). The functions of PMNs might be distinguished by their location. PMNs are frequently recruited through the oral epithelia to the oral cavity and mainly through the gingival crevice. The oral cavity is a complex environment, with many chemokines, including saliva, food residues, and over 700 colonizing bacterial species, and it is different from the nearly aseptic bloodstream (Gao et al., 2018). These substances can recruit and activate oral polymorphonuclear neutrophils (oPMNs). Under normal physiological conditions, oPMNs are responsible for eliminating the antigenic load through phagocytosis, degranulation, the release of ROS, and the formation of NETs (Landzberg et al., 2015; Fine et al., 2016).

In pathological states, PMNs will make some adaptive changes. When the host’s periodontal inflammation develops into a destructive stage, called periodontitis, the multi-aspect alterations of oPMNs are widely observed. Typically, oPMNs are recruited at a high rate and in a higher activation state (Lakschevitz et al., 2013; Landzberg et al., 2015; Nicu et al., 2018). oPMNs even have longer lives in periodontitis patients than in healthy individuals (Lakschevitz et al., 2013). Besides, a recent study showed altered gene expressions of oPMNs during oral inflammatory processes by observing the transcriptional responses of oPMNs in human experimental gingivitis models (Rijkschroeff et al., 2020). In periodontitis cases, stimulated oPMNs exhibit increased adhesion and internalization of various microorganisms and a high capacity for NET production, which is 13 times more than stimulated circulatory PMNs (Moonen et al., 2019). Besides, increased influxes, a high level of apoptosis, consistent hyperactivity, and limited killing function of oPMNs have also been observed (Nicu et al., 2018).

Like PMNs in other inflammatory conditions, not all the oPMNs in periodontitis are activated, which means only a proportion of them are functional, which has already attracted some attention. Recently, in patients with periodontitis, the proportion of CD177^+^ PMNs was significantly higher in the gingival crevicular fluid than blood. However, similar proportions of CD177^+^ PMNs were found in blood, synovial fluid, and skin chamber exudate from patients with inflammatory arthritis, indicating aseptic inflammation (Rudin et al). In a pilot study (Fine et al., 2016), researchers designed multicolor flow cytometry panels and defined three distinct neutrophil subsets based on size, granularity, and specific CD expression (cluster of differentiation) markers. These studies helped researchers further understand oPMNs and showed the potential of oPMN subsets as diagnostic and treatment monitoring biomarkers.

Over the last several years, a new perspective has been gradually accepted that although dental microbial biofilm is the initiating factor for periodontitis, the main culprits of periodontitis are uncontrolled immune responses and oxidative stresses mediated by PMNs (Sulijaya et al., 2019). In periodontitis, microorganisms and their products activate oPMNs to secrete NETs, ROS, and other factors, which can also maintain an inflammatory state and destroy the periodontium (Schauer et al., 2014; Liu et al., 2017; Sulijaya et al., 2019). Inflammatory periodontal states provide an ideal environment for the growth of some bacteria (Hajishengallis et al., 2011; Hajishengallis, 2014b), which guarantees the dominant status of pathogenic bacteria and shapes the composition of the subgingival microbiota. The vicious circle of dysbiosis and inflammation makes periodontitis persist. Therefore, it might be possible to treat periodontitis by regulating inflammation (Bartold and Van Dyke, 2017). Some studies have shown that anti-inflammatory treatments inhibit periodontitis and reverse dysbiosis (Moutsopoulos et al., 2014; Lee et al., 2016). Furthermore, the levels of periodontal tissue destruction decrease after periodontal treatment (Kaneko et al., 2018).

The homeostasis of oPMNs is critical in the oral cavity. PMNs usually provide positive effects, but they also show negative effects especially when PMN homeostasis is disrupted (Ye et al., 2011; Moutsopoulos et al., 2014; Sorensen et al., 2014; Landzberg et al., 2015; Cortes-Vieyra et al., 2016; Makkawi et al., 2017; Silva et al., 2019). Previous researches on individuals with neutrophil-related immunodeficiencies have revealed the adverse consequences of deficiencies in neutrophil counts, which seriously affect the pocket environment. Due to genetic defects, hosts with diseases such as severe congenital neutropenia (Ye et al., 2011), Papillon-Lefevre syndrome (PLS) (Sorensen et al., 2014), and leukocyte adhesion deficiency-I (Moutsopoulos et al., 2014) exhibit defective neutrophil migration, chemotaxis, adhesion, and recruitment, and persistent reductions in neutrophil counts, eventually leading to severe periodontitis (Cortes-Vieyra et al., 2016; Silva et al., 2019). Excess oPMNs contribute to the maintenance of inflammatory states, accounting for the majority of detrimental oral tissue damage by over-reactive inflammatory reactions and oxidative stresses (Makkawi et al., 2017). Researchers have determined oPMN counts by using a 30-s oral rinse obtained from healthy individuals and patients with mild, moderate, and severe late-onset periodontitis graded by periodontal parameters. The results demonstrated a correlation between oPMN counts and the severity of periodontal disease (Landzberg et al., 2015). The products of oPMNs showed a similar tendency. A retrospective case-control study revealed that in patients with RA, the serum levels of NETs were correlated positively with the values of periodontal parameters, including probing pocket depth and clinical attachment loss level (Kaneko et al., 2018). Taken together, the homeostasis of oPMNs is essential for oral health.

Overall, these findings indicate that oPMNs play a vital role in developing and maintaining periodontitis, and anti-inflammatory therapy for periodontitis is not merely symptomatic and should include and target the etiology.

## NETs in Periodontitis

### Function of NETs in Oral

As one of the defense mechanisms oPMNs, NETs released from PMNs play their role in healthy and inflammatory pocket environments. It has been demonstrated that isolated peripheral blood PMNs can be stimulated by 19 periodontal species (Hirschfeld et al., 2017). The elimination of oral bacteria is promoted by the antimicrobial properties of DNA and peptides in NETs. NETs restrict oral inflammation by clearing both pathogen-associated molecular patterns (PAMPs) and damage-associated molecular patterns (DAMPs). However, similar to systemic diseases, too many NETs can disrupt homeostasis and cause periodontitis by shielding harmful bacteria. When it comes to the adverse effects of NETs, they are associated with the periodontal pocket formation and tissue injury. Typical periodontal pockets form due to the inflammatory response. Once the periodontal pocket is formed, the NETs in the periodontal pocket, as a three-dimensional structure, wrap around the contents of the periodontal pocket, including disseminated bacteria, desquamated epithelial cells, cell debris, and biofilm matrix fragments, making it more difficult for PAMPs and DAMPs to be eliminated from the periodontal pocket. Therefore, the NET formation would be exaggerated, leading to deeper periodontal pockets. As mentioned above, NETs can also assist oPMNs to maintain periodontitis by promoting the inflammatory response. Other studies have shown that NET formation could influence tissue injury and even mortality (Yost et al., 2016; Lopes et al., 2017). Furthermore, high levels of NETs and oPMNs might contribute to oral cancer development (Jablonska et al., 2020).

### Strategies of Periodontal Pathogens

**Figure 2:**
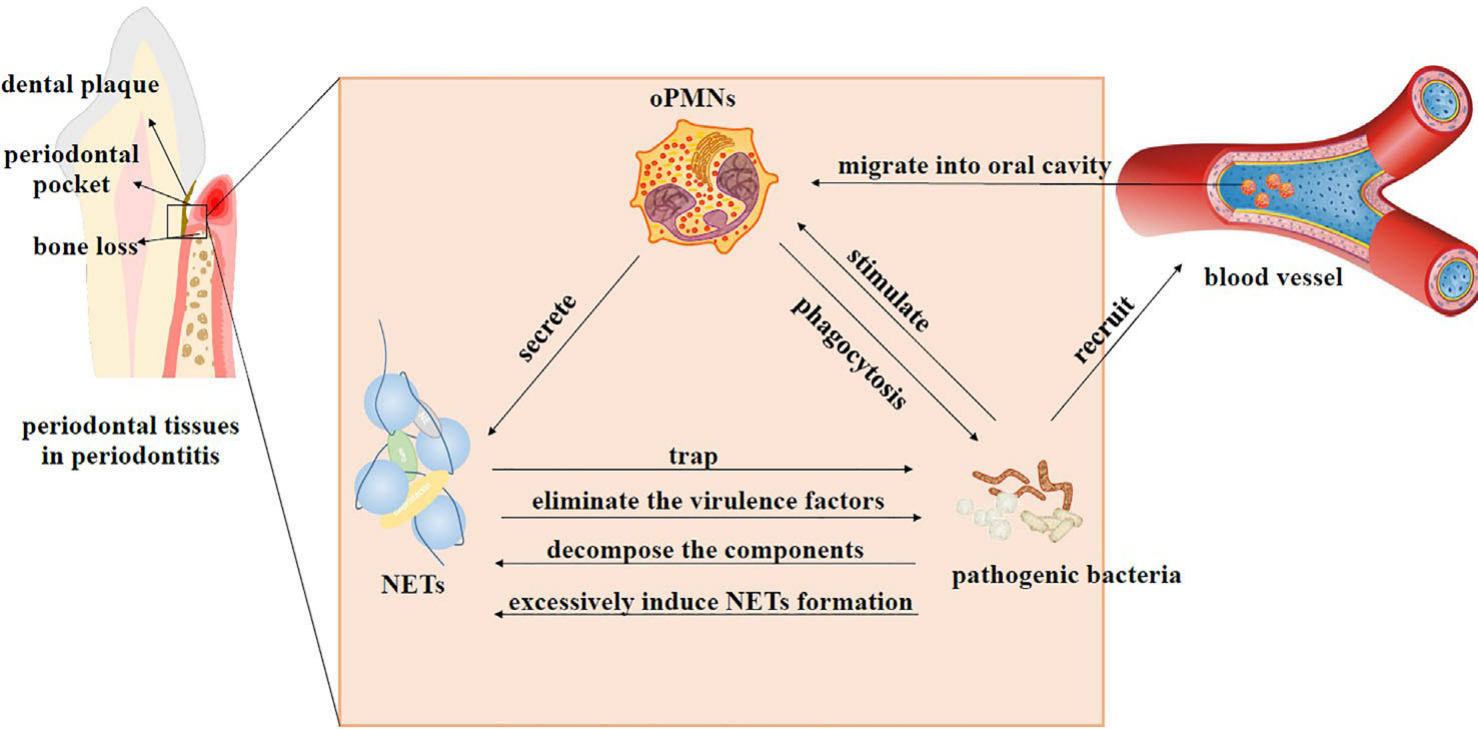
Interactions between oPMNs, NETs, and bacteria in periodontitis. In the beginning, periodontal microflora shifts from homeostasis to dysbiosis. Pathogenic bacteria recruit abundant PMNs that migrate from blood vessels into the oral cavity and become active oPMNs. Stimulated oPMNs differentiate into distinct subsets to function through different mechanisms, such as phagocytosis, degranulation, and NET formation. NETs can restrict the spread of bacteria and eliminate the virulence factors of the microorganisms. Bacteria use some techniques to deal with NETs. They can produce enzymes to decompose DNA and proteins that compose NETs. Some bacteria can strongly induce NET generation and decompose antibacterial components of NETs at the same time. High levels of NETs can benefit the dominant position of these bacteria by inhibiting others. The interaction of oPMNs, NETs, and bacteria jointly leads to excessive immune response and persistent inflammatory state, ultimately resulting in tissue injury and bone resorption.

As mentioned above, oPMNs and periodontal microorganisms jointly regulate the pocket environment to achieve homeostasis or induce dysbiosis through sophisticated signaling systems (**Figure 2**). In this dynamic process, periodontal pathogens induce NET formation (Mikolai et al., 2021), and NETs help oPMNs modulate bacteria colonizing the periodontal tissues. Periodontal bacteria have evolved mechanisms to evade the immune response of the host and even benefit from NETs (Cortes-Vieyra et al., 2016).

Concerning NETs, periodontal pathogens can produce extracellular nucleases to degrade DNA and proteases to hydrolyze antibacterial peptides. Some studies have focused on the different abilities of periodontal pathogens to avoid the antibacterial function of NETs. The DNA degradation activity of major Gram-negative periodontal pathogens, including *P. gingivalis*, *P. intermedia*, *Fusobacterium nucleatum*, and *Aggregatibacter actinomycetemcomitans*, were compared, which showed that *P. intermedia* had the highest degradation activity (Doke et al., 2017). In this study, the researchers also successfully produced recombinant proteins that can degrade DNA components of NETs with two genes selected from *P. intermedia*. One of the main virulence factors of *P. gingivalis* is *Porphyromonas* peptidylarginine deiminase (PPAD), which could convert peptidylarginine into citrulline residues and participate in the pathogenesis of RA (Potempa et al., 2017). After exposing PPAD-proficient and PPAD-deficient *P. gingivalis* to PMNs forming NETs, more PPAD-deficient bacteria were trapped in NETs and eliminated upon capture than PPAD-proficient bacteria, indicating that PPAD activity can impair NETs (Stobernack et al., 2018). Buhr et al. (de Buhr et al., 2014) concentrated on the mechanism used by *Streptococcus suis* (*S. suis*) to arthritisevade extracellular NET-mediated entrapment and killing. They finally established that *S. suis* serotype 2 could degrade NETs through the expression of *S. suis*-secreted nuclease A (SsnA). Researchers observed efficient entrapment of *S. suis* in NETs secreted by stimulated porcine PMNs through immunofluorescence microscopy. However, they also reported that *S. suis* resisted the killing of human NETs, with the involvement of SsnA in this self-protection process.

Furthermore, an isogenic SsnA mutant revealed significantly reduced NET degradation function and abortive resistance to the antimicrobial activity of NETs. Besides, *Neisseria gonorrhoeae* contains a gene encoding the presumed secretion of thermonuclease (Nuc). Under the secretion of Nuc, the bacteria exhibited a higher survival rate in the face of NETs released by PMNs (Juneau et al., 2015). Moreover, some proteases, such as LL-37, elastase, and cathepsin G (Bryzek et al., 2019), secreted by periodontal bacteria, can hydrolyze antibacterial components of NETs to reduce the bactericidal activity of NETs efficiently.

Some pathogens not only decompose the components of NETs but also achieve a dominant position in the flora by promoting host immunity to inhibit other bacteria. For example, composed with wild-type strain (W83), which strongly induces NET generation, the gingipain-null mutant strain can only mediate slightly NETs production in both aerobic and anaerobic conditions. This difference shows that gingipains are necessary for *P. gingivalis* to induce NET formation (Bryzek et al., 2019). Meanwhile, gingipains isolated from *P. gingivalis* can induce NET formation directly, while other *P. gingivalis* virulence factors, including LPS and major fimbriae, have not shown such an effect. As cysteine proteases, gingipains can also efficiently degrade NETs’ antibacterial peptides. Thus, in the subgingival plaque, excessive amounts of NETs will inhibit the reproduction of other microorganisms and create a growth-promoting environment for *P. gingivalis*. Conversely, as a major bacterium of periodontal plaque in physiological conditions, *Streptococcus oralis* can induce both the release and degradation of NETs, which might establish a balance in NET levels (Mikolai et al., 2021).

### Potential Therapeutic Agents

Based on the understanding of inflammation and NETs, researchers have found some possible therapeutic agents to reduce inflammation and tissue damage through regulating NETs.

Yost et al. (2016) found that neonatal NET-inhibitory factor (nNIF) in the umbilical cord blood can block NET formation by inhibiting key terminal events. It was also observed that additional nNIF-related peptides (NRPs) had a similar inhibitory effect. In further experiments, they demonstrated that nNIFs and NRPs could inhibit NET generation in mouse models of infection and systemic inflammation. They also provided evidence that nNIFs had beneficial effects in systemic sterile inflammation and infection models, which might have the potential as therapeutic agents. Jablonska et al. (2020) showed that flavonoids, including quercetin and luteolin, had an inhibitory effect on the NET formation in patients with oral lichen planus (OLP). Besides, DNase-1 can degrade cell-free DNA, a principal component of NETs, which mediates inflammation and coagulation. In the rat models of intestinal (Wang et al., 2018), renal (Peer et al., 2016), and hepatic (Huang et al., 2015) ischemia-reperfusion injury, the exogenous DNase-1 can accelerate the clearance of NETs and attenuate tissue damage. Doke et al. (2017) cloned the nucleases encoded by two genes selected from *Prevotella intermedia* (*P. intermedia*). Recombinant nucleases showed their potential to digest the DNA in NETs. As mentioned above, PAD4 is essential to NETs formation (Gupta et al., 2014). The commonly used pan-PAD inhibitor, Cl-amidine, and selective PAD4 inhibitors have already proved efficient *in vitro* (Lewis et al., 2015; Aliko et al., 2019). Clinically used compounds, such as sodium bicarbonate and THAM, effectively raise pH and promote the NET formation, suggesting the ability of these compounds to regulate NET levels *in vivo* (Khan et al., 2018). In addition, the adjunctive use of systemic antimicrobials, like a combination of amoxicillin and metronidazole, metronidazole alone, and azithromycin in periodontal therapy, results in statistically significant benefits in clinical outcomes (Teughels et al., 2020), which might work by transforming periodontal microbial communities from dysbiosis to homeostasis. Further studies on plaque composition analysis are helpful to understand the exact mechanism.

Inevitably, there are still some problems in the existing experiments. *In vitro* experiments, some studies have obtained oPMNs by collecting oral rinses (Rijkschroeff et al., 2020). Some others have isolated cPMNs from peripheral blood (Lewis et al., 2015; Aliko et al., 2019). Transcriptions, phenotypes, life spans, and other differences between oPMNs and cPMNs have already been demonstrated (Lakschevitz et al., 2013; Landzberg et al., 2015; Nicu et al., 2018). Further studies are necessary to determine whether or not these differences impact the generation of NETs, the reactivation of inflammation, and other aspects. Moreover, studies investigating the interaction between bacteria and neutrophils or host usually use individual microorganisms to exclude other factors. However, individual bacteria cause a specific immune response, different from the commensal microbiota (Hajishengallis et al., 2011; Mikolai et al., 2021). Exploring the combined action of multiple microorganisms might be the future direction. Besides, studies investigating inflammatory mechanisms *in vivo* have used mice models, the human experimental gingivitis models, and *Galleria mellonella* (Hajishengallis et al., 2011; Yost et al., 2016; Stobernack et al., 2018; Wang et al., 2018), which only possesses an innate immune system. Human models are rapidly inducible and reversible in nature, while it is difficult to meet the experimental needs of irreversible periodontitis because of ethical issues. Mice models can provide *in vivo* experimental environments; however, they do not guarantee whether the results can be reproduced in the human oral cavity or not.

## Conclusions and Perspectives

It has been over a decade since NETs were discovered. Although the formation and function of NETs are well understood, their specific mechanisms need to be further investigated, especially their anti-inflammatory function. In addition, ROS plays an essential role in the formation of NETs; however, the specific oxides are unknown. oPMNs actively participate in the occurrence, development, and maintenance of periodontitis, a highly prevalent disease. In this process, oPMNs secrete abundant NETs into the pocket environment to regulate bacteria and reduce tissue inflammation. On the other hand, high levels of NETs can promote an inflammatory state and provide a suitable environment for periodontal pathogenic bacteria. The dysbiotic periodontal microbial communities and the inflammatory state can be maintained by stimulating and evading host immune responses. The immune response becomes excessive, ultimately leading to periodontal tissue injury due to the interactions between periodontal microflora dysbiosis and hosts.

Some studies have shown the vital function of oPMNs and their production in maintaining periodontitis. They have also indicated that a decrease in inflammation severity might be the key to treating periodontitis. Further research is necessary to explore the specific mechanisms. Besides, there is evidence on the strategies of some periodontal bacteria to avoid NETs and survive. Based on past research, some therapeutic agents that can regulate NETs have been found, which might be a possible new approach to treat periodontitis by breaking the vicious circle of inflammation and periodontal dysbiosis.

Furthermore, as discussed above, it is necessary to investigate the techniques used to collect oPMNs, the choice of experimental models, the combined action of multiple bacterial species, the specific regulation between single bacterium and NETs, and the efficacy of potential therapeutic agents.

## Author Contributions

JW, YZ, and ML designed, wrote, and revised the manuscript. BR, LZ, and BH revised the manuscript. All authors contributed to the article and approved the submitted version.

## Funding

This study was supported by the Sichuan Science and Technology Program 2021YFH0188 (ML), National Natural Science Foundation of China grant 81400501 (ML), 81500811 (BH), and Province College Cooperation Program of Sichuan Province 2020YFSY0019 (LZ).

## Conflict of Interest

The authors declare that the research was conducted in the absence of any commercial or financial relationships that could be construed as a potential conflict of interest.

## References

[B1] Abusleme L. Dupuy A. K. Dutzan N. Silva N. Burleson J. A. Strausbaugh L. D. (2013). The subgingival microbiome in health and periodontitis and its relationship with community biomass and inflammation. ISME J. 7 (5), 1016–1025. 23303375 10.1038/ismej.2012.174 PMC3635234

[B2] Aleyd E. van Hout M. W. Ganzevles S. H. Hoeben K. A. Everts V. Bakema J. E. (2014). IgA enhances NETosis and release of neutrophil extracellular traps by polymorphonuclear cells via Fcα receptor I. J. Immunol. 192 (5), 2374–2383. 24493821 10.4049/jimmunol.1300261

[B3] Aliko A. Kaminska M. Falkowski K. Bielecka E.. (2019). Discovery of Novel Potential Reversible Peptidyl Arginine Deiminase Inhibitor. Int. J. Mol. Sci. 20 (9), 16. 10.3390/ijms20092174 PMC6539144 31052493

[B4] Amulic B. Cazalet C. Hayes G. L.. (2012). Neutrophil function: from mechanisms to disease. Annu. Rev. Immunol. 30, 459–489. 22224774 10.1146/annurev-immunol-020711-074942

[B5] Aquino-Martinez R. Rowsey J. L. Fraser D. G.. (2020). LPS-induced premature osteocyte senescence: Implications in inflammatory alveolar bone loss and periodontal disease pathogenesis. Bone 132, 115220. 31904537 10.1016/j.bone.2019.115220 PMC6990876

[B6] Bartold P. M. Van Dyke T. E. (2017). Host modulation: controlling the inflammation to control the infection. Periodontol 2000 75 (1), 317–329. 28758299 10.1111/prd.12169

[B7] Beukers N. G. van der Heijden G. J. van Wijk A. J.. (2017). Periodontitis is an independent risk indicator for atherosclerotic cardiovascular diseases among 60 174 participants in a large dental school in the Netherlands. J. Epidemiol. Community Health 71 (1), 37–42. 27502782 10.1136/jech-2015-206745 PMC5256268

[B8] Boeltz S. Amini P. Anders H. J.. (2019). To NET or not to NET:current opinions and state of the science regarding the formation of neutrophil extracellular traps. Cell Death Differ 26 (3), 395–408. 30622307 10.1038/s41418-018-0261-x PMC6370810

[B9] Branzk N. Lubojemska A. Hardison S. E.. (2014). Neutrophils sense microbe size and selectively release neutrophil extracellular traps in response to large pathogens. Nat. Immunol. 15 (11), 1017–1025. 25217981 10.1038/ni.2987 PMC4236687

[B10] Brinkmann V. Reichard U. Goosmann C.. (2004). Neutrophil extracellular traps kill bacteria. Science 303 (5663), 1532–1535. 15001782 10.1126/science.1092385

[B11] Brinkmann V. (2018). Neutrophil Extracellular Traps in the Second Decade. J. Innate Immun. 10 (5-6), 414–421. 29909412 10.1159/000489829 PMC6784051

[B12] Bryzek D. Ciaston I. Dobosz E.. (2019). Triggering NETosis via protease-activated receptor (PAR)-2 signaling as a mechanism of hijacking neutrophils function for pathogen benefits. PLoS Pathog. 15 (5), e1007773. 31107907 10.1371/journal.ppat.1007773 PMC6544335

[B13] Carrizales-Sepulveda E. F. Ordaz-Farias A. Vera-Pineda R.. (2018). Periodontal Disease, Systemic Inflammation and the Risk of Cardiovascular Disease. Heart Lung Circ. 27 (11), 1327–1334. 29903685 10.1016/j.hlc.2018.05.102

[B14] Cortes-Vieyra R. Rosales C. Uribe-Querol E. (2016). Neutrophil Functions in Periodontal Homeostasis. J. Immunol. Res. 2016. 10.1155/2016/1396106 PMC4785262 27019855

[B15] da Silva M. K. de Carvalho A. C. G. Alves E. H. P.. (2017). Genetic Factors and the Risk of Periodontitis Development: Findings from a Systematic Review Composed of 13 Studies of Meta-Analysis with 71,531 Participants. Int. J. Dent. 2017, 1914073. 28529526 10.1155/2017/1914073 PMC5424192

[B16] Daly B. Thompsell A. Sharpling J. Rooney Y. M.. (2018). Evidence summary: the relationship between oral health and dementia. Br. Dental J. 223 (11), 846–853. 10.1038/sj.bdj.2017.992 29192686

[B17] de Bont C. M. Boelens W. C. Pruijn G. J. M. (2019). NETosis, complement, and coagulation: a triangular relationship. Cell Mol. Immunol. 16 (1), 19–27. 29572545 10.1038/s41423-018-0024-0 PMC6318284

[B18] de Buhr N. Neumann A. Jerjomiceva N. von Köckritz-Blickwede M. Baums CG. (2014). Streptococcus suis DNase SsnA contributes to degradation of neutrophil extracellular traps (NETs) and evasion of NET-mediated antimicrobial activity. Microbiol. (Reading) 160 (Pt 2), 385–395. 10.1099/mic.0.072199-0 24222615

[B19] Deniset J. F. Kubes P. (2016). Recent advances in understanding neutrophils. F1000Research 5, 2912–2912. 28105328 10.12688/f1000research.9691.1 PMC5225409

[B20] Deniset J. F. Kubes P. (2018). Neutrophil heterogeneity: Bona fide subsets or polarization states? J. Leukoc. Biol. 103 (5), 829–838. 29462505 10.1002/JLB.3RI0917-361R

[B21] Denning N. L. Aziz M. Gurien S. D.. (2019). DAMPs and NETs in Sepsis. Front. Immunol. 10, 2536. 31736963 10.3389/fimmu.2019.02536 PMC6831555

[B22] Doke M. Fukamachi H. Morisaki H.. (2017). Nucleases from Prevotella intermedia can degrade neutrophil extracellular traps. Mol. Oral. Microbiol. 32 (4), 288–300. 27476978 10.1111/omi.12171 PMC5516193

[B23] Ebersole J. L. Dawson D. A. 3rd Huja E.. (2018). Age and Periodontal Health - Immunological View. Curr. Oral. Health Rep. 5 (4), 229–241. 30555774 10.1007/s40496-018-0202-2 PMC6291006

[B24] Eggert F. M. Drewell L. Bigelow J. A.. (1991). The pH of gingival crevices and periodontal pockets in children, teenagers and adults. Arch. Oral. Biol. 36 (3), 233–238. 1908671 10.1016/0003-9969(91)90091-8

[B25] Fine N. Hassanpour S. Borenstein A.. (2016). Distinct Oral Neutrophil Subsets Define Health and Periodontal Disease States. J. Dent. Res. 95 (8), 931–938. 27270666 10.1177/0022034516645564

[B26] Franck G. Mawson T. L. Folco E. J.. (2018). Roles of PAD4 and NETosis in Experimental Atherosclerosis and Arterial Injury: Implications for Superficial Erosion. Circ. Res. 123 (1), 33–42. 29572206 10.1161/CIRCRESAHA.117.312494 PMC6014872

[B27] Frangou E. Chrysanthopoulou A. Mitsios A.. (2019a). An emerging role of neutrophils and NETosis in chronic inflammation and fibrosis in systemic lupus erythematosus (SLE) and ANCA-associated vasculitides (AAV): Implications for the pathogenesis and treatment. Autoimmun Rev. 18 (8), 751–760. 31181324 10.1016/j.autrev.2019.06.011

[B28] Frangou E. Vassilopoulos D. Boletis J.. (2019b). REDD1/autophagy pathway promotes thromboinflammation and fibrosis in human systemic lupus erythematosus (SLE) through NETs decorated with tissue factor (TF) and interleukin-17A (IL-17A). Ann. Rheum Dis. 78 (2), 238–248. 30563869 10.1136/annrheumdis-2018-213181 PMC6352428

[B29] Frencken J. E. Sharma P. Stenhouse L.. (2017). Global epidemiology of dental caries and severe periodontitis - a comprehensive review. J. Clin. Periodontol 44 (Suppl 18), S94–S105. 28266116 10.1111/jcpe.12677

[B30] Fuchs T. A. Abed U. Goosmann C.. (2007). Novel cell death program leads to neutrophil extracellular traps. J. Cell Biol. 176 (2), 231–241. 17210947 10.1083/jcb.200606027 PMC2063942

[B31] Galluzzi L. Vitale I. Aaronson S. A.. (2018). Molecular mechanisms of cell death: recommendations of the Nomenclature Committee on Cell Death 2018. Cell Death Differ 25 (3), 486–541. 29362479 10.1038/s41418-017-0012-4 PMC5864239

[B32] Gao L. Xu T. S. Huang G.. (2018). Oral microbiomes: more and more importance in oral cavity and whole body. Protein Cell 9 (5), 488–500. 29736705 10.1007/s13238-018-0548-1 PMC5960472

[B33] Gillenius E. Urban C. F. (2015). The adhesive protein invasin of Yersinia pseudotuberculosis induces neutrophil extracellular traps via β1 integrins. Microbes Infect. 17 (5), 327–336. 25576025 10.1016/j.micinf.2014.12.014

[B34] Gupta A. K. Giaglis S. Hasler P.. (2014). Efficient neutrophil extracellular trap induction requires mobilization of both intracellular and extracellular calcium pools and is modulated by cyclosporine A. PLoS One 9 (5), e97088. 24819773 10.1371/journal.pone.0097088 PMC4018253

[B35] Hahn J. Schauer C. Czegley C.. (2019). Aggregated neutrophil extracellular traps resolve inflammation by proteolysis of cytokines and chemokines and protection from antiproteases. FASEB J. 33 (1), 1401–1414. 30130433 10.1096/fj.201800752R PMC6355082

[B36] Haider P. Kral-Pointner J. B. Mayer J.. (2020). Neutrophil Extracellular Trap Degradation by Differently Polarized Macrophage Subsets. Arterioscler. Thromb. Vasc. Biol. 40 (9), 2265–2278. 32673525 10.1161/ATVBAHA.120.314883 PMC7447175

[B37] Hajishengallis G. Liang S. Payne M. A.. (2011). Low-Abundance Biofilm Species Orchestrates Inflammatory Periodontal Disease through the Commensal Microbiota and Complement. Cell Host Microbe 10 (5), 497–506. 22036469 10.1016/j.chom.2011.10.006 PMC3221781

[B38] Hajishengallis G. (2014a). Immunomicrobial pathogenesis of periodontitis: keystones, pathobionts, and host response. Trends Immunol. 35 (1), 3–11. 24269668 10.1016/j.it.2013.09.001 PMC3947349

[B39] Hajishengallis G. (2014b). The inflammophilic character of the periodontitis-associated microbiota. Mol. Oral. Microbiol. 29 (6), 248–257. 24976068 10.1111/omi.12065 PMC4232466

[B40] Hajishengallis G. (2015). Periodontitis: from microbial immune subversion to systemic inflammation. Nat. Rev. Immunol. 15 (1), 30–44. 25534621 10.1038/nri3785 PMC4276050

[B41] Halverson T. W. Wilton M. Poon K. K.. (2015). DNA is an antimicrobial component of neutrophil extracellular traps. PLoS Pathog. 11 (1), e1004593. 25590621 10.1371/journal.ppat.1004593 PMC4295883

[B42] Hirschfeld J. Dommisch H. Skora P.. (2015). Neutrophil extracellular trap formation in supragingival biofilms. Int. J. Med. Microbiol. 305 (4-5), 453–463. 25959370 10.1016/j.ijmm.2015.04.002

[B43] Hirschfeld J. White P. C. Milward M. R.. (2017). Modulation of Neutrophil Extracellular Trap and Reactive Oxygen Species Release by Periodontal Bacteria. Infect. Immun. 85 (12). 10.1128/IAI.00297-17 PMC5695129 28947649

[B44] Hosseinzadeh A. Thompson P. R. Segal B. H.. (2016). Nicotine induces neutrophil extracellular traps. J. Leukoc. Biol. 100 (5), 1105–1112. 27312847 10.1189/jlb.3AB0815-379RR PMC5069087

[B45] Huang H. Tohme S. Al-Khafaji A. B.. (2015). Damage-associated molecular pattern-activated neutrophil extracellular trap exacerbates sterile inflammatory liver injury. Hepatology 62 (2), 600–614. 25855125 10.1002/hep.27841 PMC4515210

[B46] Jablonska E. Garley M. Surazynski A.. (2020). Neutrophil extracellular traps (NETs) formation induced by TGF-beta in oral lichen planus - Possible implications for the development of oral cancer. Immunobiology 225 (2), 151901. 31882256 10.1016/j.imbio.2019.151901

[B47] Jiménez-Alcázar M. Rangaswamy C. Panda R.. (2017). Host DNases prevent vascular occlusion by neutrophil extracellular traps. Science 358 (6367), 1202–1206. 29191910 10.1126/science.aam8897

[B48] Johnson M. B. Criss A. K. (2013). Neisseria gonorrhoeae phagosomes delay fusion with primary granules to enhance bacterial survival inside human neutrophils. Cell Microbiol. 15 (8), 1323–1340. 23374609 10.1111/cmi.12117 PMC3713093

[B49] Juneau R. A. Stevens J. S. Apicella M. A.. (2015). A thermonuclease of Neisseria gonorrhoeae enhances bacterial escape from killing by neutrophil extracellular traps. J. Infect. Dis. 212 (2), 316–324. 25605868 10.1093/infdis/jiv031 PMC4490236

[B50] Kaneko C. Kobayashi T. Ito S.. (2018). Circulating levels of carbamylated protein and neutrophil extracellular traps are associated with periodontitis severity in patients with rheumatoid arthritis: A pilot case-control study. PLoS One 13 (2), e0192365. 29394286 10.1371/journal.pone.0192365 PMC5796721

[B51] Kenny E. F. Herzig A. Kruger R.. (2017). Diverse stimuli engage different neutrophil extracellular trap pathways. Elife 6. 10.7554/eLife.24437 PMC5496738 28574339

[B52] Khan M. A. Philip L. M. Cheung G.. (2018). Regulating NETosis: Increasing pH Promotes NADPH Oxidase-Dependent NETosis. Front. Med. (Lausanne) 5, 19. 29487850 10.3389/fmed.2018.00019 PMC5816902

[B53] Kilian M. Chapple I. L. C. Hannig M.. (2016). The oral microbiome - an update for oral healthcare professionals. Br. Dental J. 221 (10), 657–666. 10.1038/sj.bdj.2016.865 27857087

[B54] Kimball A. S. Obi A. T. Diaz J. A.. (2016). The Emerging Role of NETs in Venous Thrombosis and Immunothrombosis. Front. Immunol. 7, 236. 27446071 10.3389/fimmu.2016.00236 PMC4921471

[B55] Knight J. S. Zhao W. Luo W.. (2013). Peptidylarginine deiminase inhibition is immunomodulatory and vasculoprotective in murine lupus. J. Clin. Invest. 123 (7), 2981–2993. 23722903 10.1172/JCI67390 PMC3696545

[B56] Knodler L. A. Vallance B. A. Celli J.. (2010). Dissemination of invasive Salmonella via bacterial-induced extrusion of mucosal epithelia. Proc. Natl. Acad. Sci. U. S. A. 107 (41), 17733–17738. 20876119 10.1073/pnas.1006098107 PMC2955089

[B57] Kolaczkowska E. Jenne C. N. Surewaard B. G.. (2015). Molecular mechanisms of NET formation and degradation revealed by intravital imaging in the liver vasculature. Nat. Commun. 6, 6673. 25809117 10.1038/ncomms7673 PMC4389265

[B58] Konig M. F. Abusleme L. Reinholdt J.. (2016). Aggregatibacter actinomycetemcomitans-induced hypercitrullination links periodontal infection to autoimmunity in rheumatoid arthritis. Sci. Trans. Med. 8 (369), 12. 10.1126/scitranslmed.aaj1921 PMC5384717 27974664

[B59] Kubes P. (2018). The enigmatic neutrophil: what we do not know. Cell Tissue Res. 371 (3), 399–406. 29404726 10.1007/s00441-018-2790-5

[B60] Lakschevitz F. S. Aboodi G. M. Glogauer M. (2013). Oral neutrophil transcriptome changes result in a pro-survival phenotype in periodontal diseases. PLoS One 8 (7), e68983. 23874838 10.1371/journal.pone.0068983 PMC3708893

[B61] Lamont R. J. Hajishengallis G. (2015). Polymicrobial synergy and dysbiosis in inflammatory disease. Trends Mol. Med. 21 (3), 172–183. 25498392 10.1016/j.molmed.2014.11.004 PMC4352384

[B62] Lamont R. J. Koo H. Hajishengallis G. (2018). The oral microbiota: dynamic communities and host interactions. Nat. Rev. Microbiol. 16 (12), 745–759. 30301974 10.1038/s41579-018-0089-x PMC6278837

[B63] Landzberg M. Doering H. Aboodi G. M.. (2015). Quantifying oral inflammatory load: oral neutrophil counts in periodontal health and disease. J. Periodontal Res. 50 (3), 330–336. 25040400 10.1111/jre.12211

[B64] Lazzaretto B. Fadeel B. (2019). Intra- and Extracellular Degradation of Neutrophil Extracellular Traps by Macrophages and Dendritic Cells. J. Immunol. 203 (8), 2276–2290. 31519860 10.4049/jimmunol.1800159 PMC6778307

[B65] Lee C. T. Teles R. Kantarci A.. (2016). Resolvin E1 Reverses Experimental Periodontitis and Dysbiosis. J. Immunol. 197 (7), 2796–2806. 27543615 10.4049/jimmunol.1600859 PMC5026932

[B66] Leech M. T. Bartold P. M. (2015). The association between rheumatoid arthritis and periodontitis. Best Pract. Res. Clin. Rheumatol. 29 (2), 189–201. 26362738 10.1016/j.berh.2015.03.001

[B67] Lewis H. D. Liddle J. Coote J. E.. (2015). Inhibition of PAD4 activity is sufficient to disrupt mouse and human NET formation. Nat. Chem. Biol. 11 (3), 189–191. 25622091 10.1038/nchembio.1735 PMC4397581

[B68] Li T. Zhang Z. Li X.. (2020). Neutrophil Extracellular Traps: Signaling Properties and Disease Relevance. Mediators Inflamm 2020, 9254087. 32774152 10.1155/2020/9254087 PMC7407020

[B69] Liu C. Mo L. Niu Y.. (2017). The Role of Reactive Oxygen Species and Autophagy in Periodontitis and Their Potential Linkage. Front. Physiol. 8. 10.3389/fphys.2017.00439 PMC5481360 28690552

[B70] Loe H. Theilade E. Jensen S. B. (1965). Experimental Gingivitis in Man. J. Periodontol 36 (3), 177–187. 14296927 10.1902/jop.1965.36.3.177

[B71] Lopes D. E. M. Jabr C. L. Dejani N. N.. (2017). Inhibition of 5-Lipoxygenase (5-Lo) Attenuates Inflammation and Bone Resorption in Lipopolysaccharide (Lps)-Induced Periodontal Disease. J. Periodontol, 1–18. 10.1902/jop.2017.170210 28871891

[B72] Magan-Fernandez A. O'Valle F. Abadia-Molina F.. (2019). Characterization and comparison of neutrophil extracellular traps in gingival samples of periodontitis and gingivitis: A pilot study. J. Periodontal Res. 54 (3), 218–224. 30298590 10.1111/jre.12621

[B73] Magan-Fernandez A. Rasheed Al-Bakri S. M. O'Valle F.. (2020). Neutrophil Extracellular Traps in Periodontitis. Cells 9 (6). 10.3390/cells9061494 PMC7349145 32575367

[B74] Makkawi H. Hoch S. Burns E.. (2017). Porphyromonas gingivalis Stimulates TLR2-PI3K Signaling to Escape Immune Clearance and Induce Bone Resorption Independently of MyD88. Front. Cell. Infect Microbiol. 7. 10.3389/fcimb.2017.00359 PMC5550410 28848717

[B75] Martinelli S. Urosevic M. Daryadel A.. (2004). Induction of genes mediating interferon-dependent extracellular trap formation during neutrophil differentiation. J. Biol. Chem. 279 (42), 44123–44132. 15302890 10.1074/jbc.M405883200

[B76] Metzler K. D. Goosmann C. Lubojemska A.. (2014). A myeloperoxidase-containing complex regulates neutrophil elastase release and actin dynamics during NETosis. Cell Rep. 8 (3), 883–896. 25066128 10.1016/j.celrep.2014.06.044 PMC4471680

[B77] Mikolai C. Branitzki-Heinemann K. Ingendoh-Tsakmakidis A.. (2021). Neutrophils exhibit an individual response to different oral bacterial biofilms. J. Oral. Microbiol. 13 (1). 10.1080/20002297.2020.1856565 PMC7733916 33391628

[B78] Miyoshi A. Yamada M. Shida H.. (2016). Circulating Neutrophil Extracellular Trap Levels in Well-Controlled Type 2 Diabetes and Pathway Involved in Their Formation Induced by High-Dose Glucose. Pathobiology 83 (5), 243–251. 27189166 10.1159/000444881

[B79] Moonen C. G. J. Hirschfeld J. Cheng L.. (2019). Oral Neutrophils Characterized: Chemotactic, Phagocytic, and Neutrophil Extracellular Trap (NET) Formation Properties. Front. Immunol. 10, 635. 30984197 10.3389/fimmu.2019.00635 PMC6449731

[B80] Moonen C. G. Buurma K. G. Faruque M. R.. (2020). Periodontal therapy increases neutrophil extracellular trap degradation. Innate Immun. 26 (5), 331–340. 31757174 10.1177/1753425919889392 PMC7903525

[B81] Moutsopoulos N. M. Konkel J. Sarmadi M.. (2014). Defective Neutrophil Recruitment in Leukocyte Adhesion Deficiency Type I Disease Causes Local IL-17-Driven Inflammatory Bone Loss. Sci. Trans. Med. 6 (229), 11. 10.1126/scitranslmed.3007696 PMC4090608 24670684

[B82] Nicu E. A. Rijkschroeff P. Wartewig E.. (2018). Characterization of oral polymorphonuclear neutrophils in periodontitis patients: a case-control study. BMC Oral. Health 18, 9. 30143044 10.1186/s12903-018-0615-2 PMC6109268

[B83] Nociti F. H. Casati M. Z. Duarte P. M. (2015). Current perspective of the impact of smoking on the progression and treatment of periodontitis. Periodontol 2000 67 (1), 187–210. 25494601 10.1111/prd.12063

[B84] Obama T. Ohinata H. Takaki T.. (2019). Cooperative Action of Oxidized Low-Density Lipoproteins and Neutrophils on Endothelial Inflammatory Responses Through Neutrophil Extracellular Trap Formation. Front. Immunol. 10, 1899. 31447863 10.3389/fimmu.2019.01899 PMC6696608

[B85] Papayannopoulos V. (2018). Neutrophil extracellular traps in immunity and disease. Nat. Rev. Immunol. 18 (2), 134–147. 28990587 10.1038/nri.2017.105

[B86] Peer V. Abu Hamad R. Berman S.. (2016). Renoprotective Effects of DNAse-I Treatment in a Rat Model of Ischemia/Reperfusion-Induced Acute Kidney Injury. Am. J. Nephrol. 43 (3), 195–205. 27073834 10.1159/000445546

[B87] Pieterse E. Jeremic I. Czegley C.. (2016). Blood-borne phagocytes internalize urate microaggregates and prevent intravascular NETosis by urate crystals. Sci. Rep. 6, 38229. 27917897 10.1038/srep38229 PMC5137018

[B88] Pihlstrom B. L. Michalowicz B. S. Johnson N. W. (2005). Periodontal diseases. Lancet 366 (9499), 1809–1820. 16298220 10.1016/S0140-6736(05)67728-8

[B89] Pilsczek F. H. Salina D. Poon K. K.. (2010). A novel mechanism of rapid nuclear neutrophil extracellular trap formation in response to Staphylococcus aureus. J. Immunol. 185 (12), 7413–7425. 21098229 10.4049/jimmunol.1000675

[B90] Pires R. H. Felix S. B. Delcea M. (2016). The architecture of neutrophil extracellular traps investigated by atomic force microscopy. Nanoscale 8 (29), 14193–14202. 27387552 10.1039/c6nr03416k

[B91] Potempa J. Mydel P. Koziel J. (2017). The case for periodontitis in the pathogenesis of rheumatoid arthritis. Nat. Rev. Rheumatol 13 (10), 606–620. 28835673 10.1038/nrrheum.2017.132

[B92] Rijkschroeff P. Schoenmaker T. Caspers M.. (2020). Dentistry and OMICS: Transcriptome Dynamics of an Oral Ecosystem as Measured by Changes in Oral Polymorphonuclear Neutrophils in Experimental Gingivitis. Omics-a J. Integr. Biol. 24 (9), 531–540. 10.1089/omi.2020.0034 32559408

[B93] Rodriguez-Espinosa O. Rojas-Espinosa O. Moreno-Altamirano M. M.. (2015). Metabolic requirements for neutrophil extracellular traps formation. Immunology 145 (2), 213–224. 25545227 10.1111/imm.12437 PMC4427386

[B94] Rosier B. T. De Jager M. Zaura E.. (2014). Historical and contemporary hypotheses on the development of oral diseases: are we there yet? Front. Cell. Infect Microbiol. 4. 10.3389/fcimb.2014.00092 PMC4100321 25077073

[B95] Rosier B. T. Marsh P. D. Mira A. (2018). Resilience of the Oral Microbiota in Health: Mechanisms That Prevent Dysbiosis. J. Dental Res. 97 (4), 371–380. 10.1177/0022034517742139 29195050

[B96] Rudin A. D. Amirbeagi F. Davidsson L.. The neutrophil subset defined by CD177 expression is preferentially recruited to gingival crevicular fluid in periodontitis. J. Leukocyte Biol., 14. 10.1002/JLB.3A0520-081RR 32531826

[B97] Sanz M. Del Castillo A. M. Jepsen S.. (2020). Periodontitis and Cardiovascular Diseases. Consensus Report. Global Heart 15 (1), 1. 32489774 10.5334/gh.400 PMC7218770

[B98] Schauer C. Janko C. Munoz L. E.. (2014). Aggregated neutrophil extracellular traps limit inflammation by degrading cytokines and chemokines. Nat. Med. 20 (5), 511–517. 24784231 10.1038/nm.3547

[B99] Sierra J. M. Fuste E. Rabanal F.. (2017). An overview of antimicrobial peptides and the latest advances in their development. Expert Opin. Biol. Ther. 17 (6), 663–676. 28368216 10.1080/14712598.2017.1315402

[B100] Silva L. M. Brenchley L. Moutsopoulos N. M. (2019). Primary immunodeficiencies reveal the essential role of tissue neutrophils in periodontitis. Immunol Rev. 287 (1), 226–235. 30565245 10.1111/imr.12724 PMC7015146

[B101] Socransky S. S. Haffajee A. D. (2005). Periodontal microbial ecology. Periodontol 2000 38, 135–187. 15853940 10.1111/j.1600-0757.2005.00107.x

[B102] Sorensen O. E. Borregaard N. (2016). Neutrophil extracellular traps - the dark side of neutrophils. J. Clin. Invest. 126 (5), 1612–1620. 27135878 10.1172/JCI84538 PMC4855925

[B103] Sorensen O. E. Clemmensen S. N. Dahl S. L.. (2014). Papillon-Lefevre syndrome patient reveals species-dependent requirements for neutrophil defenses. J. Clin. Invest. 124 (10), 4539–4548. 25244098 10.1172/JCI76009 PMC4191054

[B104] Spaan A. N. Surewaard B. G. Nijland R.. (2013). Neutrophils versus Staphylococcus aureus: a biological tug of war. Annu. Rev. Microbiol. 67, 629–650. 23834243 10.1146/annurev-micro-092412-155746

[B105] Stobernack T. Espina M. d. T. Mulder L. M.. (2018). A Secreted Bacterial Peptidylarginine Deiminase Can Neutralize Human Innate Immune Defenses. Mbio 9 (5). 10.1128/mBio.01704-18 PMC6212822 30377277

[B106] Sulijaya B. Takahashi N. Yamazaki K. (2019). Host modulation therapy using anti-inflammatory and antioxidant agents in periodontitis: A review to a clinical translation. Arch. Oral. Biol. 105, 72–80. 31288144 10.1016/j.archoralbio.2019.07.002

[B107] Teughels W. Feres M. Oud V.. (2020). Adjunctive effect of systemic antimicrobials in periodontitis therapy: A systematic review and meta-analysis. J. Clin. Periodontol 47, 257–281. 10.1111/jcpe.13264 31994207

[B108] Tonetti M. S. Bottenberg P. Conrads G.. (2017). Dental caries and periodontal diseases in the ageing population: call to action to protect and enhance oral health and well-being as an essential component of healthy ageing - Consensus report of group 4 of the joint EFP/ORCA workshop on the boundaries between caries and periodontal diseases. J. Clin. Periodontol 44 (Suppl 18), S135–S144. 28266112 10.1111/jcpe.12681

[B109] Ussar S. Fujisaka S. Kahn C. R. (2016). Interactions between host genetics and gut microbiome in diabetes and metabolic syndrome. Mol. Metab. 5 (9), 795–803. 27617202 10.1016/j.molmet.2016.07.004 PMC5004229

[B110] Vitkov L. Klappacher M. Hannig M.. (2009). Extracellular neutrophil traps in periodontitis. J. Periodontal Res. 44 (5), 664–672. 19453857 10.1111/j.1600-0765.2008.01175.x

[B111] von Kockritz-Blickwede M. Goldmann O. Thulin P.. (2008). Phagocytosis-independent antimicrobial activity of mast cells by means of extracellular trap formation. Blood 111 (6), 3070–3080. 18182576 10.1182/blood-2007-07-104018

[B112] Wang S. K. Xie T. Sun S. L.. (2018). DNase-1 Treatment Exerts Protective Effects in a Rat Model of Intestinal Ischemia-Reperfusion Injury. Sci. Rep. 8, 9. 30542063 10.1038/s41598-018-36198-2 PMC6290768

[B113] Welin A. Amirbeagi F. Christenson K.. (2013). The human neutrophil subsets defined by the presence or absence of OLFM4 both transmigrate into tissue in vivo and give rise to distinct NETs in vitro. PLoS One 8 (7), e69575. 23922742 10.1371/journal.pone.0069575 PMC3726694

[B114] White P. Sakellari D. Roberts H.. (2016). Peripheral blood neutrophil extracellular trap production and degradation in chronic periodontitis. J. Clin. Periodontol 43 (12), 1041–1049. 27678376 10.1111/jcpe.12628

[B115] White P. C. Hirschfeld J Milward M. R. Cooper P. R.. (2018). Cigarette smoke modifies neutrophil chemotaxis, neutrophil extracellular trap formation and inflammatory response-related gene expression. J. Periodontal Res. 53 (4), 525–535. 29574730 10.1111/jre.12542

[B116] Ye Y. Wondimu G. Fahlen B.. (2011). Mutations in the ELANE Gene are Associated with Development of Periodontitis in Patients with Severe Congenital Neutropenia. J. Clin. Immunol. 31 (6), 936–945. 21796505 10.1007/s10875-011-9572-0 PMC3223588

[B117] Yost C. C. Schwertz H. Cody M. J.. (2016). Neonatal NET-inhibitory factor and related peptides inhibit neutrophil extracellular trap formation. J. Clin. Invest. 126 (10), 3783–3798. 27599294 10.1172/JCI83873 PMC5096809

[B118] Yousefi S. Gold J. A. Andina N.. (2008). Catapult-like release of mitochondrial DNA by eosinophils contributes to antibacterial defense. Nat. Med. 14 (9), 949–953. 18690244 10.1038/nm.1855

[B119] Yousefi S. Mihalache C. Kozlowski E.. (2009). Viable neutrophils release mitochondrial DNA to form neutrophil extracellular traps. Cell Death Differ 16 (11), 1438–1444. 19609275 10.1038/cdd.2009.96

[B120] Yuen J. Pluthero F. G. Douda D. N.. (2016). NETosing Neutrophils Activate Complement Both on Their Own NETs and Bacteria via Alternative and Non-alternative Pathways. Front. Immunol. 7, 137. 27148258 10.3389/fimmu.2016.00137 PMC4831636

[B121] Zhang X. Zhuchenko O. Kuspa A.. (2016). Social amoebae trap and kill bacteria by casting DNA nets. Nat. Commun. 7, 10938. 26927887 10.1038/ncomms10938 PMC4773522

